# A novel small RNA is important for biofilm formation and pathogenicity in *Pseudomonas aeruginosa*

**DOI:** 10.1371/journal.pone.0182582

**Published:** 2017-08-03

**Authors:** Patrick K. Taylor, Antonius T. M. Van Kessel, Antonio Colavita, Robert E. W. Hancock, Thien-Fah Mah

**Affiliations:** 1 Department of Biochemistry, Microbiology and Immunology, University of Ottawa, Ottawa, Ontario, Canada; 2 Neuroscience Program, Ottawa Hospital Research Institute, Ottawa, Ontario, Canada; 3 Department of Microbiology and Immunology, University of British Columbia, Vancouver, British Columbia, Canada; East Carolina University Brody School of Medicine, UNITED STATES

## Abstract

The regulation of biofilm development requires multiple mechanisms and pathways, but it is not fully understood how these are integrated. Small RNA post-transcriptional regulators are a strong candidate as a regulatory mechanism of biofilm formation. More than 200 small RNAs in the *P*. *aeruginosa* genome have been characterized in the literature to date; however, little is known about their biological roles in the cell. Here we describe the identification of the novel regulatory small RNA, SrbA. This locus was up-regulated 45-fold in *P*. *aeruginosa* strain PA14 biofilm cultures. Loss of SrbA expression in a deletion strain resulted in a 66% reduction in biofilm mass. Furthermore, the mortality rate over 72 hours in *C*. *elegans* infections was reduced to 39% when infected with the *srbA* deletion strain compared to 78% mortality when infected with the parental wild-type *P*. *aeruginosa* strain. There was no significant effect on culture growth or adherence to surfaces with loss of SrbA expression. Also loss of SrbA expression had no effect on antibiotic resistance to ciprofloxacin, gentamicin, and tobramycin. We conclude that SrbA is important for biofilm formation and full pathogenicity of *P*. *aeruginosa*.

## Introduction

Bacterial biofilms are aggregated communities of cells that are embedded within a self-produced extracellular matrix [1,2]. The matrix can contain various biopolymers including polysaccharides, DNA, and protein [3–7]; it enables structured association of cells within the biofilm, mediates tight adhesion to surfaces, and promotes the mechanical stability of biofilms. The matrix also helps to maintain an internal environment and entrap extracellular degradative enzymes [8]. While biofilm colonies undergo dispersal to spread cells into the environment, they are intrinsically resilient and difficult to disrupt [9,10]. The resilience and resistance to treatment of biofilms poses a continual challenge in clinical settings when treating bacterial infections and decontaminating equipment [11]. Biofilm infections are significantly more adaptively resistant to antibiotics due primarily to their altered gene expression patterns and in part due to the protection provided by the extracellular matrix [12–14]. In addition, biofilms contaminating surfaces are difficult to fully sterilize or remove mechanically, creating reservoirs of pathogenic bacteria in hospitals [15,16].

*P*. *aeruginosa* is a Gram-negative γ-Proteobacteria and is a significant opportunistic pathogen in individuals with compromised immune systems and natural barriers. Individuals with the genetic condition cystic fibrosis are highly susceptible to having their airways colonized by environmental sources of *P*. *aeruginosa* [17]. *P*. *aeruginosa* is highly adaptable to the host environment and is capable of altering regulatory networks to enable survival during chronic infections.

It has become increasingly appreciated that complex regulatory mechanisms govern biofilm development and enable both major and subtle responses that are dependent on cues from the external environment. Recent studies on pathogenic bacteria have demonstrated that non-coding, small RNA (sRNA) transcripts have important effects on biofilm formation and virulence in a host [18–21]. Recent studies into the transcriptome of *P*. *aeruginosa* uncovered novel sRNAs that are expressed under conditions that replicate aspects of the host environment during infections and biofilm development [22–24]. Many studies have catalogued novel sRNAs, however, there have been few studies that characterize their biological roles and determine the importance of sRNA-mediated regulation in complex adaptive modes of growth such as biofilm formation.

A particular category of sRNAs, called trans-sRNAs, are encoded as independent genes and usually do not form part of an operon [25]. These are often around 50–400 nucleotides in length [26,27] and act as post-transcriptional regulators of protein synthesis acting through short stretches (5–7 nucleotides) of base-pairing complementarity with target mRNAs to either promote or inhibit translation [27]. Trans-sRNAs are of particular interest when studying regulation of complex activities like biofilm formation, because such trans-sRNAs can have a high number of mRNA interaction targets throughout the genome, leading to broad post-transcriptional regulation. Typically these sRNAs interact at or near the ribosomal binding site (RBS) of an mRNA transcript [27]. Through interaction with the target mRNA, an sRNA may have a negative regulatory effect by blocking the ribosome or a positive regulatory effect by altering secondary structures through binding to the target mRNA and making the RBS available [25,27]. Trans-sRNAs can also exert their regulation through affecting mRNA stability. An sRNA can bind to its target and recruit RNases that will degrade an mRNA target [25]. Regulation by sRNAs can affect highly complex and diverse expression networks as well as providing cross-talk between signalling networks.

Having a better understanding of the biofilm lifestyle and regulation is of significant importance to developing new treatments for bacterial biofilm infections that comprise two thirds of all infections. Here we describe the novel **s**RNA **r**egulator of **b**iofilms **A**, SrbA that is important for biofilm formation and pathogenesis in *P*. *aeruginosa*. Biofilms grown under laboratory conditions were significantly diminished in an *srbA* deletion strain. Using a *Caenorhabditis elegans* model of infection, it was also found that the *srbA* deletion strain displayed a significant reduction in pathogenicity.

## Materials and methods

### Bacterial strain generation and growth conditions

Strains and plasmids used in this study are listed in Table 1. All primers used in this study are listed in S1 Table. In the wild-type strain of *P*. *aeruginosa* UCBPP-PA14, a chromosomal deletion mutant of the sRNA gene locus was generated by allelic exchange [28]. Two 1 kb fragments flanking *srbA* were amplified using PCR. The two flanking fragments were digested with BamHI and ligated. The ligated 2 kbp deletion fragment and pEX18Gm suicide vector were digested with EcoRI and SalI before being ligated together to generate the pEXΔ*srbA* construct. The deletion construct was first introduced into *Escherichia coli* by heat-shock. *P*. *aeruginosa* UCBPP-PA14 was transformed with the deletion construct through incubation with the conjugative transfer strain S17-1. Transconjugants were isolated by growth on LB agar plates containing 15 μg/ml gentamicin to select for PA14 cells carrying the pEXΔ*srbA* plasmid and 30 μg/ml nalidixic acid to eliminate *E*. *coli* S17-1 cells. Strains containing the chromosomal deletion were confirmed by PCR as well as by sequencing at the StemCore facility of the Ottawa Hospital Research Institute.

**Table 1:** Strains and plasmids used in this study.

	Description^*a*^	Source
**Strains**		
UCBPP-PA14	*P*. *aeruginosa* PA14 wild-type strain	[29]
Δ*srbA*	UCBPP-PA14 containing a chromosomal deletion of *srbA*	This study
*srbA*^+^	Δ*srbA* background complemented with pUC*srbA*, Cb^R^	This study
PA14 *srbA*^+^	UCBPP-PA14 background complemented with pUC*srbA*, Cb^R^	This study
DH5α	*E*. *coli* λ^-^, φ80*lacZ*ΔM15, F^-^, Δ(*lacZYA*-*argF*)*U169*, *endA1*, *gyr*A96, *hsdR17*(r_k_^-^, m_k_^+^), *phoA*, *recA1*, *relA1*, *supE44*, *thi-1*	[30]
S17-1	*E*. *coli* λ*pir*, RP4-Tc::Mu Km::Tn7, *hsdR*^*-*^, *hsdM*^*+*^, *pro*, *recA*, *thi*, Sm^R^, Tp^R^	[31]
OP50	*E*. *coli* uracil auxotroph, Sm^R^	[32], [33]
**Plasmids**		
pEX18Gm	Gene replacement vector, *oriT*^*+*^, *sacB*^*+*^, MCS from pUC18, Gm^R^	[34]
pEXΔ*srbA*	pEX18Gm carrying a 2kb insertion in the MCS consisting of flanking regions but lacking *srbA*, Gm^R^	This study
pUCP18	Cloning and expression vector for use in *E*. *coli* and *P*. *aeruginosa*, MCS from pUC18, c*olE1*^+^, *ori1600*^*+*^, Ap^R^ (*E*. *coli*)/Cb^R^ (*P*. *aeruginosa*)	[35]
pUC*srbA*	pUCP18 with insertion of *srbA* in the MCS, Cb^R^	This study

*a*. Abbreviations: Ap^R^, ampicillin resistance; Cb^R^, carbenicillin resistance; Gm^R^, gentamicin resistance; Sm^R^, streptomycin resistance; Tp^R^, trimethoprim resistance; MCS, multiple cloning site.

To enable complementation by *srbA* expression, the entire 239 bp region was cloned into the expression plasmid pUCP18. PA14 was transformed by electroporation as described previously using 5 ms pulses at 2.5 kV in 0.2 cm electroporation cuvettes [36]. PA14 transformants carrying the pUC*srbA* construct were selected on LB agar with 100 μg/ml carbenicillin. Successful transformation of the plasmid was confirmed by restriction enzyme digestion and visualization on an agarose gel. For most experiments, cultures were first grown overnight in LB medium at 37°C. For growth assays (described below) 1% tryptone and M63 minimal media were used in addition to LB. When used, M63 basal medium at pH 7 consisted of final concentrations of 1x M63 salts (22 mM KH_2_PO_4_, 40 mM K_2_HPO_4_, 15 mM (NH_4_)_2_SO_4_), 0.4% (w/v) L-arginine, and 1 mM MgSO_4_.

### RNA isolation and quantitative PCR

Whole cell RNA was isolated from biofilm and planktonic cultures. Colony biofilms were grown by spotting multiples of 5 μl of overnight cultures onto M63 agar plates. Inoculated plates were incubated at 37°C for 24 h plus an additional 24 h at room temperature before harvesting. Planktonic cultures were grown by inoculating a 1/100 dilution of overnight cultures into 3 ml M63 medium and incubating at 37°C with shaking for 4 h or until an OD_600_ of between 0.3–0.5 was reached. RNA isolation was performed by pelleting re-suspended biofilm colonies or planktonic cultures and incubating cells in 1 ml of TRIzol® from Thermo Fisher Scientific, Inc. for 5 min with regular pipetting to homogenize the samples. RNA was then purified using the PureLink® RNA Minikit according to instructions from Thermo Fisher Scientific, Inc. with an additional DNase digestion step before the final isolation of RNA. RNA was tested for DNA contamination by PCR. cDNA was generated using the iScript™ kit from Bio-Rad Laboratories, Inc. For each sample, 0.7 μg of RNA was used to synthesis cDNA. Quantitative PCR (RT-qPCR) was performed using the MyIQ™ system from Bio-Rad Laboratories, Inc. and fold-changes in expression were calculated by the ΔΔCt method using *rpoD* as a reference gene. Primers used in this study are listed in S1 Table.

### Crystal violet staining assays

Static biofilms were grown according to previously established protocols in 96-well microtitre plates [37]. Overnight cultures were diluted 1/100 into fresh LB containing a final concentration of 100 μg/ml carbenicillin and 100 μl aliquoted into each well with several aliquots tested for each strain and biological replicate. Static biofilms were grown for 24 h at 37°C, after which the microtitre plates were washed twice and each well was loaded with 100 μl of 0.1% (w/v) crystal violet. Plates were incubated at room temperature for 20 minutes before washing twice. Biofilm formation was quantified by solubilizing the crystal violet stain in 110 μl per well of 70% (v/v) ethanol. Absorbance was read at 595 nm to measure relative differences in biofilm biomass.

### Growth curves

Growth studies were performed by inoculating 100 ml of fresh medium in an Erlenmeyer flask with overnight cultures at a 1/100 dilution. Inoculated flasks were then placed on a shaker at 140 rpm. Samples were taken immediately after inoculation and every 30 min to assess optical density at 600 nm (OD_600_).

### Minimal inhibitory concentrations (MICs) and minimal bactericidal concentrations (MBC) for planktonic cultures

Assaying for MICs was performed by serial dilution in a microtitre plate as described previously [38]. Two-fold serial dilutions of ciprofloxacin, gentamicin, and tobramycin were prepared and innoculated with overnight cultures of *P*. *aeruginosa* strains and MICs were taken after incubating at 37°C for 18 hours. The MIC was taken as the first well in the microtitre plate to have no observable growth. The MBCs for planktonic cultures- were assayed by spotting 5 μl of culture from MIC assay microtitre plates starting at the MIC followed by increasing concentrations onto LB agar plates with no antibiotic added. The MBC was taken as the first spotted culture to have no colonies growing.

### Slow killing model in *C*. *elegans*

Slow killing plates were prepared using sterile nematode growth medium as described previously [32,39,40] and consisted of 0.2% (w/v) bacto-agar, 0.25% (w/v) peptone, 50 mM NaCl, 25 mM KH_2_PO_4,_ 1 mM CaCl_2_, 1 mM MgSO_4_, and adjusted to pH 6. A volume of 100 μl of overnight cultures of *P*. *aeruginosa* strains or *E*. *coli* OP50 were spread on plates and grown as a lawn overnight at 37°C. Two technical replicates were performed for each biological replicate and a total of 3 biological replicates were performed. For each technical replicate, a total of 30 synchronized L4 stage *C*. *elegans* were picked and seeded onto plates containing *P*. *aeruginosa* or *E*. *coli* and left at room temperature for 72 hours. Counts of dead worms were taken every 24 hours. A worm was considered dead if unresponsive to touch.

### Bioinformatic analyses

Sequence complementarity searches and alignments were performed using the BLASTn and TargetRNA2 servers [41–43]. The SrbA transcript sequence was queried limiting the searches to the UCBPP-PA14 genome. Alignments returned were then manually checked to determine if the SrbA transcript does have a complementary alignment to the expressed mRNA transcript of the potential interaction sequence. Regulator motif searches and database comparisons were carried out using the PRODORIC database [44]. Searches for protein coding regions were performed using ExPASy [45]. Multiple sequence alignments were performed using Clustal Omega [46].

## Results

### The sRNA SrbA was highly up-regulated in *P*. *aeruginosa* biofilms

**Fig 1:**
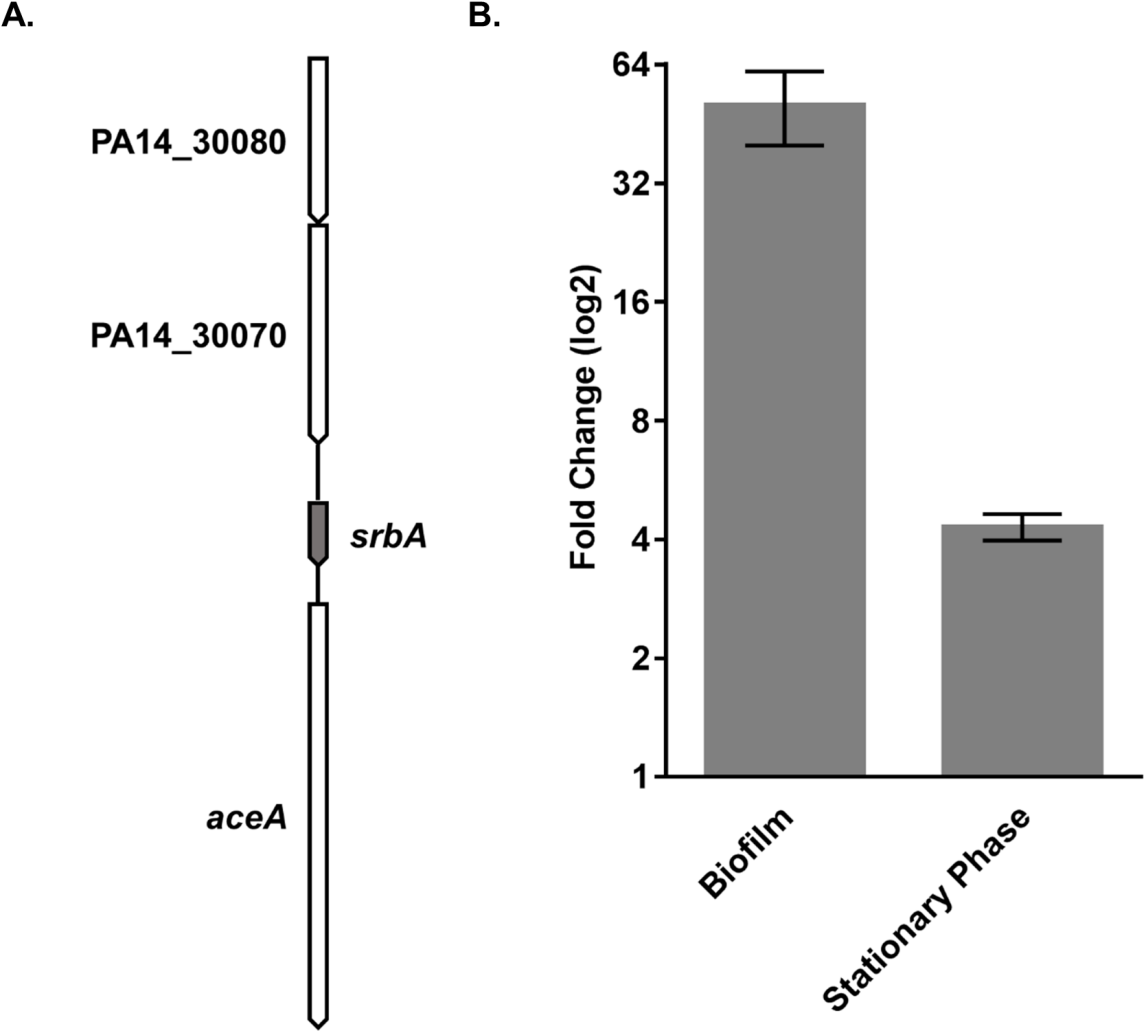
Expression of SrbA under biofilm and stationary conditions. **A)** Schematic representation of the local genetic region where *srbA* is encoded. The uncharacterized genes have been labelled with PA14 designations, PA14_30070 and PA14_30080. **B)** RT-qPCR measuring the expression of SrbA was performed using whole cell RNA purified from wild type PA14 that was grown as a biofilm or a stationary growth phase planktonic culture. Fold changes (relative to exponential growth phase planktonic cells) represented (log2 scale) are the mean of 3 biological replicates and error bars are standard error of the mean.

SrbA was previously identified in independent published studies characterizing the *P*. *aeruginosa* transcriptome using second-generation sequencing methods [22–24]. These previous studies referred to SrbA as PA2633.1 [22], pant235 [23], and PA14sr_067 [24]. Using transcriptomic data available through the *Pseudomonas* Genome Database [37] and the *Pseudomonas* Browser [24], we determined that the gene *srbA* was encoded in an intergenic region on the reverse strand of the UCBPP-PA14 genome. *srbA* is 239 bp in length and encoded from nucleotides 2,604,298 through to 2,604,536 in an intergenic region with no presence of operons between isocitrate lyase *aceA* and an uncharacterized gene [22–24,46] (Fig 1A). There are no rho-independent terminators present in *srbA*. The *Pseudomonas* Genome Database, in addition to BLASTn searches, indicated that the sequence of *srbA* was conserved in all sequenced strains of *P*. *aeruginosa* [42,47].

Expression of SrbA in biofilm and planktonic cultures was assessed by RT-qPCR. SrbA was found to be up-regulated by 45-fold during biofilm growth compared to planktonic exponential growth phase cultures (Fig 1B). Furthermore, SrbA was 4-fold up-regulated in planktonic stationary phase growth cultures relative to exponential phase growth cultures (Fig 1B).

Given the possibility that sRNAs might express short peptides that are biologically active, it was important to check if there was any recognizable peptide coding sequence within SrbA. The gene lacked a recognizable Shine-Delgarno sequence upstream of any potential start codon found in the sequence and indeed in silico translation of the SrbA transcript did not reveal any prospective expressed peptide sequences in any reading frame on either strand [24,45].

### The *srbA* deletion strain had a significantly reduced ability to form biofilms

**Fig 2:**
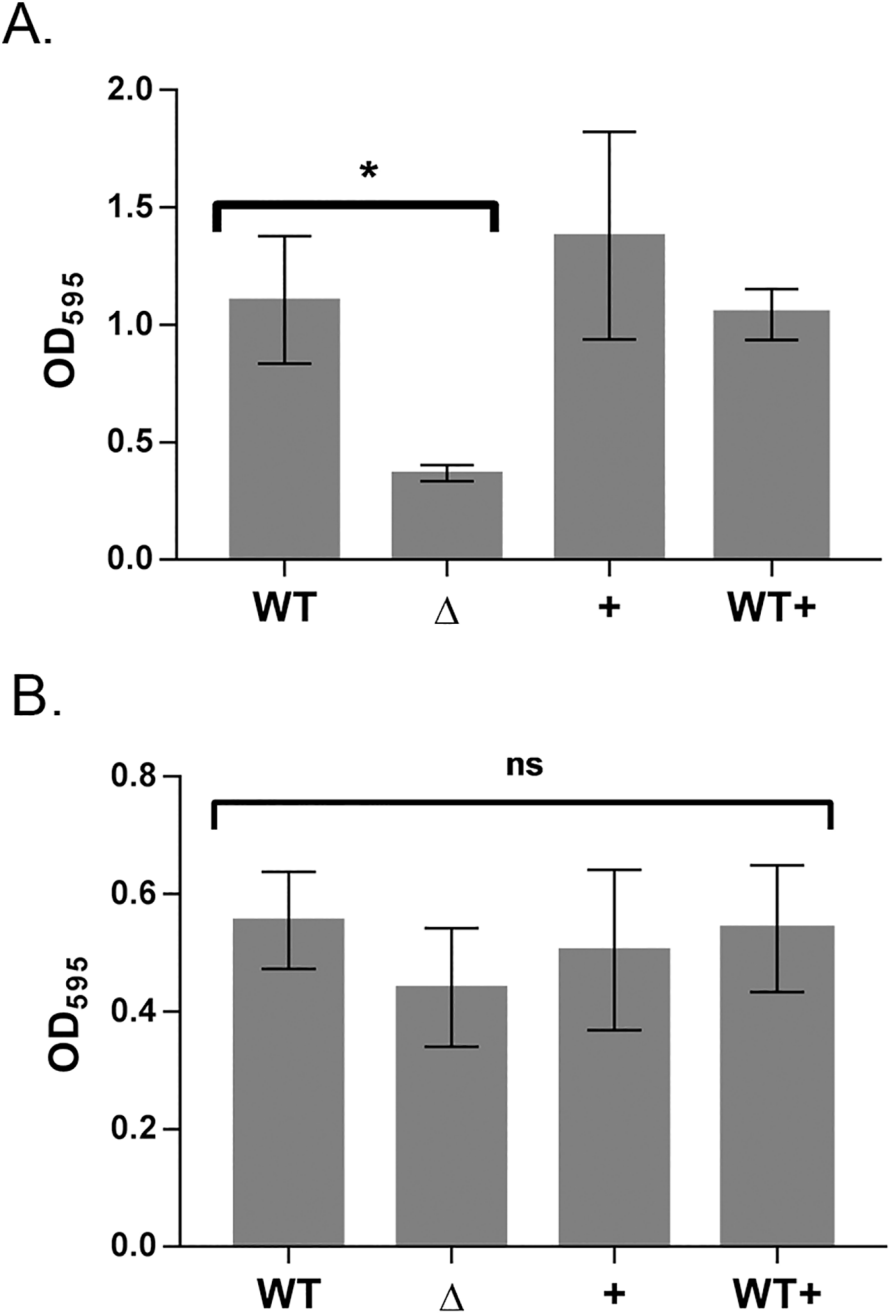
Effect of *srbA* on biofilm formation. **A)** Static biofilms were grown for 24 h in microtitre plates for the parental PA14 (WT), Δ*srbA* strain (Δ), SrbA complementation strain carrying the pUC*srbA* plasmid (+), and PA14 transformed with pUC*srbA* (WT+). After 24 h biofilm cultures stained with 0.1% crystal violet and absorbance at 595 nm was taken. A student’s t test was performed to determine significance. * represents p < 0.05. **B)** An assessment of rapid attachment for early biofilm formation was performed by incubating mid-log phase cultures for 30 min at room temperature in a microtitre plate before staining with crystal violet for cells attached to wells. WT and Δ*srbA* were transformed with empty pUCP18 plasmid and all strains were grown in 100 μg/ml carbenicillin. A one-way ANOVA was performed to determine no significance (ns). Both graphs represent the results of 4 biological replicates and error bars represent the standard error.

A *srbA* deletion mutant strain was constructed in order to carry out phenotypic analysis of the mutant. Deletion of *srbA* was confirmed through sequencing, PCR amplification of the gene locus on the chromosome (S1 Fig), and loss of expression of SrbA through use of RT-qPCR (S2 Fig). It was also determined that there were no polar effects on expression of *aceA*, the gene immediately downstream of *srbA* (S3 Fig). Since SrbA was highly up-regulated under biofilm conditions, the ability of a strain carrying a deletion of *srbA* to form biofilms was tested by using a static biofilm formation model. The deletion mutant strain produced only 34% the amount of biofilm (p < 0.05) compared to the wild-type strain (Fig 2A).

Rapid attachment assays were performed to determine if there was a deficiency in the ability of Δ*srbA* to adhere to surfaces [48–50]. In contrast to its deficiency in biofilm formation, Δ*srbA* demonstrated no significant reduction in its ability to adhere to surfaces (Fig 2B).

Complementing the mutant by expressing SrbA from the pUC*srbA* plasmid restored wild-type levels of biofilm formation (Fig 2A) and had no added effect on rapid attachment (Fig 2B). Additionally, the wild-type strain overexpressing SrbA from the pUC*srbA* plasmid displayed no alteration in biofilm phenotype.

### Deletion of *srbA* had no impact on growth or antibiotic resistance in *P*. *aeruginosa*

**Fig 3:**
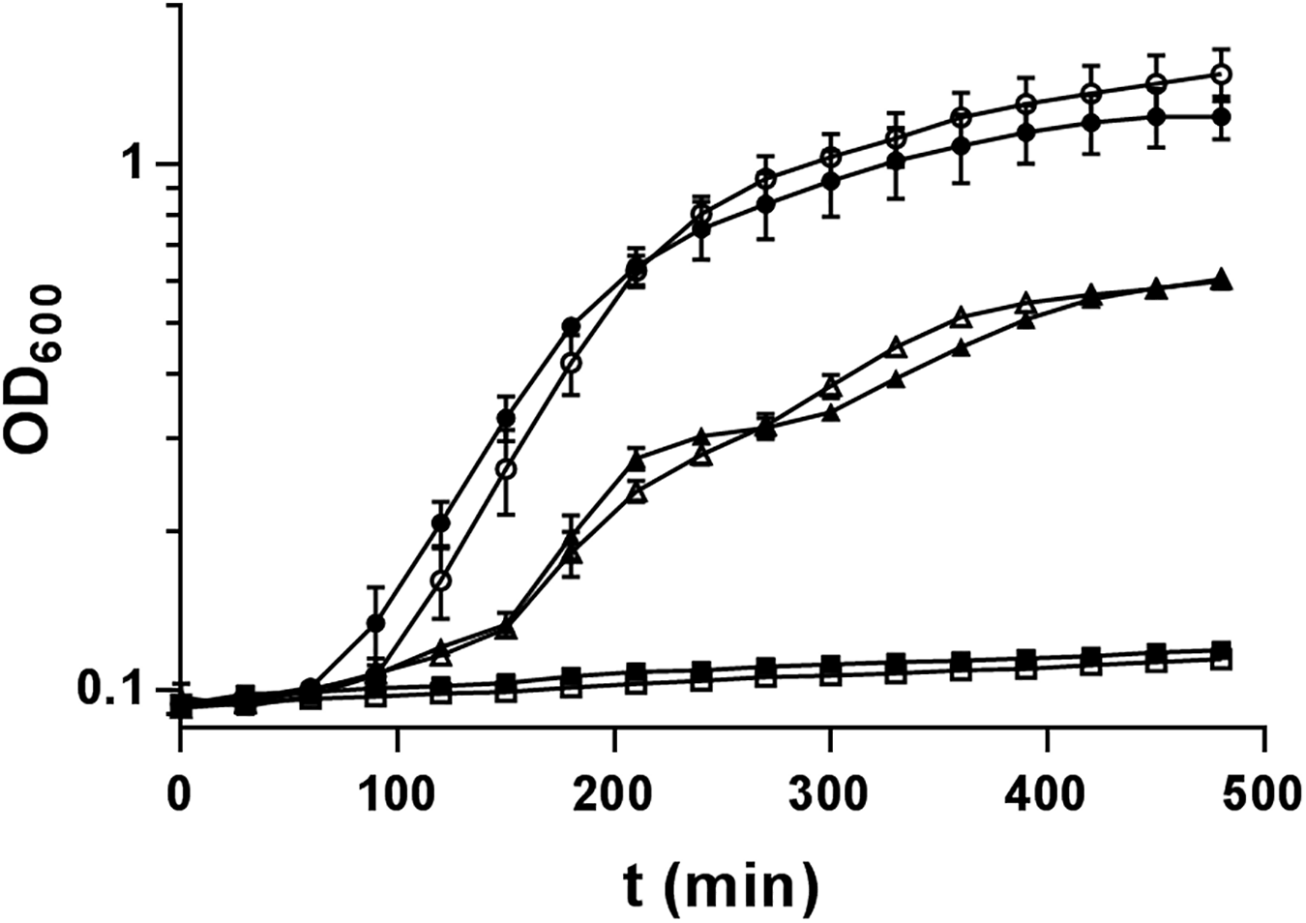
Characterization of growth phenotypes in the Δ*srbA* mutant. Growth studies were performed as an assessment of the fitness of the PA14 wild type (solid fill data points) or the Δ*srbA* mutant (open data points). Growth studies were performed using the rich medium LB (circles), and minimal media 1% tryptone (triangles) and M63 (squares). Data represented are the mean and error bars are the standard error of the mean of 3 biological replicates.

To determine if the biofilm deficiency was due to generally depressed cell health as a result of mutagenesis, growth and antibiotic resistance of the *srbA* deletion mutant were assayed. Δ*srbA* had no growth deficiency in defined minimal medium and rich medium when compared to the wild-type strain (Fig 3). Furthermore, the susceptibility phenotype to three clinically relevant antibiotics (ciprofloxacin, gentamicin, and tobramycin) was tested. The *srbA* deletion strain showed no significant change in resistance to any of these antibiotics compared to the wild-type strain (Table 2).

**Table 2:** Minimal inhibitory concentrations (MICs) and minimal bactericidal concentration in planktonic cultures (MBCs) to antibiotics.

	MIC (μg/ml)^*a*^		MBC (μg/ml)^*a*^	
Antibiotic	WT^*b*^	Δ*srbA*^*b*^	WT^*b*^	Δ*srbA*^*b*^
Ciprofloxacin	0.1	0.05	0.8	0.8
Gentamicin	2	2	16	16
Tobramycin	2	2	16	16

*a*. Data listed are the mode of 5 biological replicates.

*b*. “WT” represents UCBPP-PA14 parental strain and “Δ*srbA*” is the *srbA* deletion strain.

### The *srbA* deletion strain was attenuated in a *C*. *elegans* slow killing model

**Fig 4:**
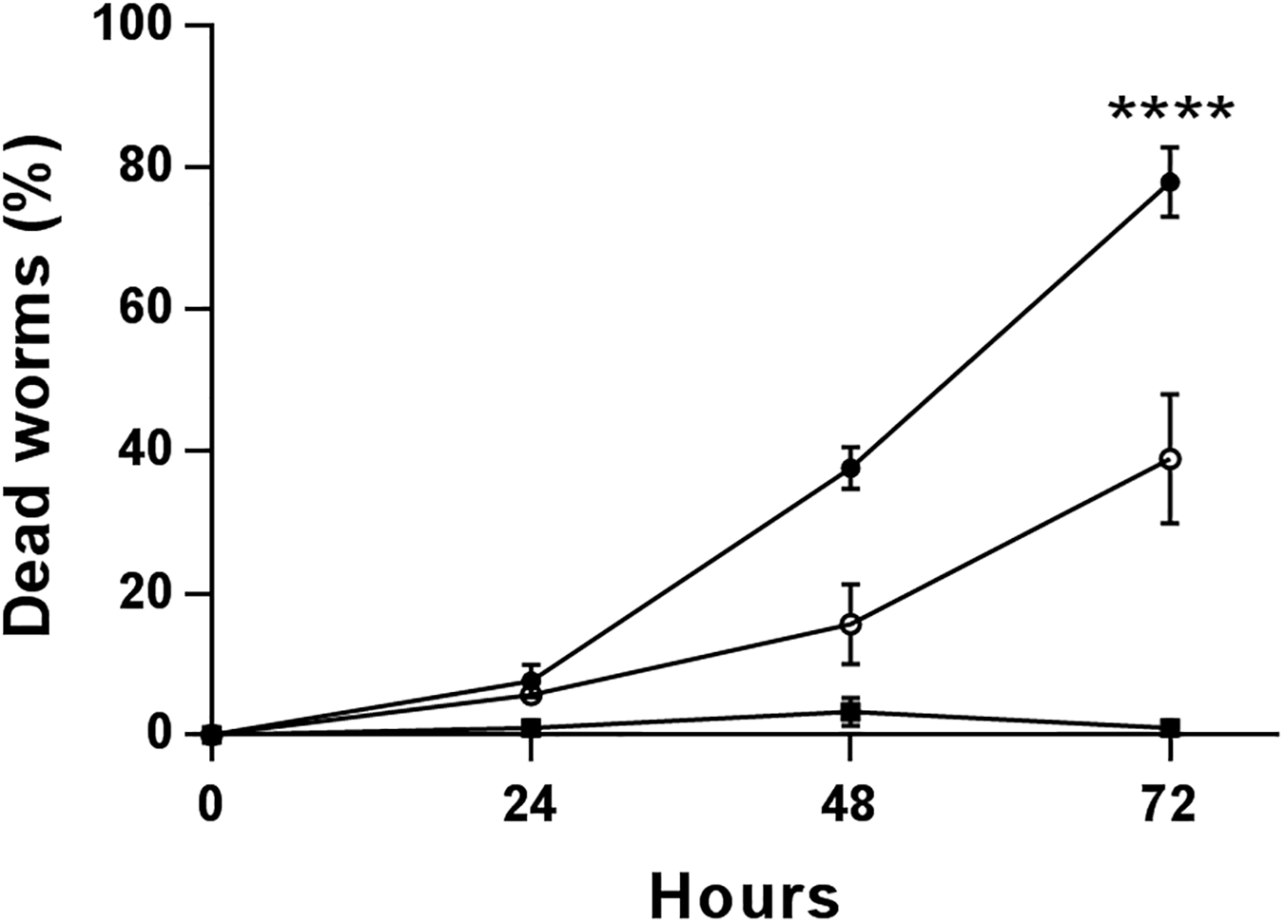
Killing of *C*. *elegans* infected with *P*. *aeruginosa* under slow killing conditions. *C*. *elegans* fed on a lawn of bacteria for 72 hours and mortality was calculated from counting dead worms. The PA14 wild type and Δ*srbA* mutant are represented by circles, solid and open, respectively. The normal food for *C*. *elegans*, *E*. *coli* OP50, was used as a negative control. Error bars are the standard error of the mean for 3 biological replicates. Student’s t test was used to determine statistical significance at the 72 hour time point. **** represents p < 0.0001.

The ability to form biofilms can contribute to the ability of a pathogen to persist within its host. Thus, it was hypothesized that the biofilm deficiency observed in the *srbA* deletion strain would result in reduced virulence and persistence in an animal host. We used a slow-killing model system to assay the ability of *P*. *aeruginosa* to persist in *C*. *elegans* that is considered a biofilm infection model of *Pseudomonas* [32,50,51]. After 72 hours, *C*. *elegans* infected with the *srbA* deletion strain displayed a 39% mortality rate that was significantly reduced compared to the 78% mortality rate observed in worms infected with the wild-type strain (Fig 4).

### The *srbA* has complementarity with sixty-one putative mRNA targets

**Fig 5:**
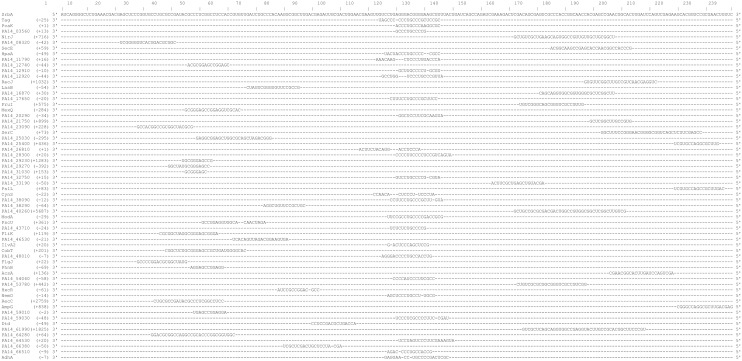
Multiple sequence alignment of SrbA with stretches of complementarity in mRNA transcripts. Clustal Omega was utilized to generate an alignment in FASTA format that is presented here in a linear layout. Transcript length is indicated by the numbers in the top row. The 5’ to 3’ orientation of sequences are provided. The bracketed numbers indicate where the 5’ end of the complementary sequence is relative to the translational start site of the putative mRNA target. If no gene name exists the PA14 gene designation is provided.

The reduced biofilm phenotype of the *srbA* deletion strain could be due to multiple factors being affected by loss of *srbA*. Trans-sRNAs often have large numbers of targets on which they exert their effects [26,27]. sRNAs typically act on their targets through short stretches of complementarity to mRNA transcripts either resulting in an inhibition or enhancement of translation through mechanisms including altering the availability of the RBS or affecting mRNA stability via recruitment of RNase E [25,26,27,52]. A search was performed using the entire SrbA transcript sequence to query against the entire *P*. *aeruginosa* PA14 genome using both TargetRNA2 and BLASTn. TargetRNA2 specifically searches for complementarity of the 5’ UTR of a putative target. Putative targets manually selected using BLAST searches were chosen on the basis that interactions with targets can occur outside of the 5’ UTR for sRNA mechanisms affecting mRNA stability. A multiple sequence alignment of these putative targets was also generated (Fig 5). To determine whether the loss of *srbA* had any effect, the expression of the 61 putative mRNA targets was assessed by RT-qPCR. It was found that the transcript levels of 26 of the putative mRNA targets were increased (2 by ≥4-fold, 7 more by ≥2-fold) or decreased (12 by ≥4-fold and 5 more by 2-fold) by greater than two-fold in the *srbA* deletion strain compared to the parental PA14 wildtype grown as biofilms (Table 3).

**Table 3:** Genes from the *P*. *aeruginosa* UCBPP-PA14 genome that have short sequence complementarity with SrbA and that were tested for transcript levels in the *srbA* mutant.

Gene Annotation	Gene Name	Gene Function	Fold Change Difference^*a*^
**Metabolism**			
PA14_00110	*tag*	DNA-3-methyladenine glycosidase I	-1.81 ±0.21
PA14_06670	*nirJ*	Heme d1 biosynthesis	+6.64 ±6.56
PA14_11000	*hpaA*	4-Hydroxyphenylacetate 3-monooxygenase large chain	+2.17 ±1.56
PA14_21750		Putative acetyltransferase	-1.38 ±0.33
PA14_23090		Putative 2-Keto-3-deoxy-6-phosphogluconate aldolase	-1.90 ±0.09
PA14_23270	*serC*	3-Phosphoserine aminotransferase	-2.16 ±0.09
PA14_25400		Putative phosphodiesterase	-3.25 ±0.15
PA14_37965	*cynS*	Cyanate hydratase	+1.06 ±0.33
PA14_38090		Putative pseudouridylate synthase	+1.33 ±0.71
PA14_47100	*ilvA2*	Threonine dehydrastase	+1.83 ±0.88
PA14_47670	*cobT*	Cobalamin biosynthesis	+2.58 ±1.85
PA14_48010		Putative semialdehyde dehydrogenase	+2.82 ±2.69
PA14_51350	*phnB*	Anthranilate synthase component II	-1.39 ±0.22
PA14_52800	*acsA*	Acetyl-coenzyme A synthetase	+1.52 ±0.60
PA14_54040		Putative amino acid permease	+1.08 ±0.58
PA14_55580	*nemO*	Heme oxygenase	-8.94 ±0.02
PA14_60100	*dtd*	Deoxycytidine triphosphate deaminase	-1.82 ±0.24
PA14_68670		Putative carboxypeptidase	-2.03 ±0.35
PA14_71630	*adhA*	Alcohol dehydrogenase	-3.64 ±0.11
Transport and Secretion			
PA14_02900	*pcaK*	4-Hydroxybenzoate transporter	-1.17 ±0.66
PA14_08695	*secE*	Protein secretion across cytoplasmic membrane	+7.82 ±5.74
PA14_11790		Putative amino acid transporter	-1.90 ±0.27
PA14_12920		Putative taurine ABC transporter periplasmic protein	+1.00 ±0.59
PA14_16870		Probable ATP-binding component of ABC transporter	-1.46 ±0.45
PA14_18250	*fruI*	Phosphotransferase system transporter	-3.11 ±0.16
PA14_25020		Probable ATP-binding component of ABC transporter	-1.23 ±0.21
PA14_31030		Putative cation efflux system protein	-1.43 ±0.29
PA14_40390	*modA*	Molybdate binding precursor	+1.75 ±1.38
PA14_53780		Probable major facilitator superfamily transporter	-2.29 ±0.37
PA14_55440	*hxcR*	Type II secretion system protein	-5.72 ±0.13
PA14_64280		Probable permease of ABC transporter	-5.37 ±0.17
PA14_66380		Putative potassium/proton antiporter	-4.19 ±0.12
Virulence Associated Factors			
PA14_16250	*lasB*	Metalloproteinase	+2.99 ±2.29
PA14_35600	*pslL*	Exopolysaccharide synthesis	-1.29 ±0.09
PA14_42660	*pscU*	Translocation protein in type III secretion	-1.82 ±0.38
PA14_45830	*fliK*	Flagellar hook-length control	-1.19 ±1.63
PA14_50380	*flgJ*	Flagellar structural component	+1.18 ±1.13
Antibiotic Resistance Associated Factors			
PA14_18780	*mexQ*	RND efflux transporter	-1.95 ±0.47
PA14_57100	*ampG*	Permease signal transducer involved in β-lactam resistance	-2.74 ±0.18
Regulation and Signaling			
PA14_26810		Putative two-component sensor	-1.10 ±0.49
PA14_29260		Probable transcriptional regulator	+1.02 ±0.15
PA14_40260		Probable transcriptional regulator	+1.54 ±0.75
PA14_43710		Putative methyl-accepting chemotaxis transducer	+1.56 ±0.43
PA14_66510		Putative MFS transporter	-1.60 ±0.57
Genetic Maintenance and Repair			
PA14_16220	*recJ*	Single-stranded DNA specific exonuclease	-2.06 ±0.14
PA14_20290		Putative DNA binding protein	+2.03 ±0.48
PA14_55690	*recC*	Exodeoxyribonuclease V gamma chain	-4.31 ±0.12
Unknown Function, Hypothetical Protein			
PA14_03560			-4.52 ±0.13
PA14_08310			-1.07 ±0.31
PA14_12740			+1.08 ±0.45
PA14_12910			-1.67 ±0.38
PA14_17650			-1.75 ±0.38
PA14_28300			-4.17 ±0.03
PA14_29230			-4.85 ±0.11
PA14_32750			-10.63 ±0.07
PA14_33190			-1.13 ±0.66
PA14_38290			+1.29 ±1.09
PA14_46530			+2.38 ±1.17
PA14_59010			-1.79 ±0.26
PA14_61990			-16.80 ±0.04
PA14_64530			+1.01 ±0.67

***a*.** Results from RT-qPCR are presented as the linear fold-change difference of transcript levels in the *srbA* deletion strain compared to the parental PA14 wildtype grown as biofilms. Values are the mean of 3 biological repeats and the standard error of the mean.

## Discussion

This study aimed to characterize the biological role of the novel sRNA SrbA. This sRNA was found to be highly upregulated relative to other sRNAs under biofilm conditions in *P*. *aeruginosa* (Fig 1B). It was determined that Δ*srbA* was highly reduced in its ability to develop as a biofilm compared to the wild-type strain and restoring expression of SrbA from a plasmid was sufficient to restore wild-type levels of biofilm formation (Fig 2A). It was also found that this biofilm deficiency is not due to the *srbA* deletion strain having an inability to adhere to surfaces and establish biofilms (Fig 2B) or any growth deficiency of the mutant strain (Fig 3). Additionally, no effect on antibiotic resistance was observed in the *srbA* deletion strain (Table 2). Taken together, these data demonstrate that the SrbA has an important role for the biofilm mode of growth beyond the attachment step.

Use of the *C*. *elegans* slow-killing model demonstrated that there is greater survival of *C*. *elegans* when infected with the *srbA* deletion strain compared to the wild-type strain. During an infection with *P*. *aeruginosa*, the host mounts an innate immune response through neutrophil activity that is of particular interest due to the tissue damage that occurs through inflammation [53]. *C*. *elegans* possesses an ancestral analogue of our innate immune system and is an applicable model to study host-pathogen interactions in *P*. *aeruginosa* infections [32]. This slow killing model assesses the ability of *P*. *aeruginosa* to develop and persist as a biofilm being the major contributor to pathogenesis [32,51,54]. It is therefore reasonable to conclude that the attenuated phenotype of the *srbA* deletion strain is due to its reduced ability to develop a biofilm.

Through use of TargetRNA2 and BLAST searches, 61 putative targets in the genome were identified that were within open reading frames and where the sequence complementarity was evident between the sRNA and mRNA transcripts (Table 3). The alignment of the complementary regions of putative targets with SrbA demonstrates that there is a region of SrbA within the span of nucleotides 120–150 where there is greater complementarity (24 of 61 putative targets) than in any other region of SrbA (Fig 5). Such a primary seed region for complementarity is common for sRNAs. However, the putative targets with complementarity in this region did not share any other known features such as gene function, interaction site in the 5’ UTR, biological role, region of the genome, etc.

Of the 61 putative mRNA targets of SrbA, there were 26 putative targets that displayed changes greater than 2-fold in transcript levels (Table 3). While sRNAs tend to affect their target gene expression at the level of protein stability, they are also known to exert effects on mRNA transcript stability [25,27]. Binding of an sRNA to its mRNA target can act to promote stability of a transcript or it may encourage degradation through recruitment of RNase E. Therefore, it is likely that the significant changes observed in 26 of the putative mRNA targets of SrbA in the *srbA* deletion strain are due to a loss of SrbA affecting regulation of stability and degradation. Additionally, of these 26 putative targets there are 9 genes (PA14_03560, *hpaA*, PA14_26810, PA14_32750, *ilvA2*, PA14_48010, *nemO*, PA14_59030, and *adhA*) that have complementarity in the region of nucleotides 120–150 where there was found to be a greater concentration of alignment of targets with SrbA (Fig 5). Future work with purified transcripts and RNase E could be performed to validate this.

The remaining 35 putative mRNA targets that did not display any significant change in transcript levels are likely under SrbA regulation through another mechanism such as affecting availability of the RBS. Future work could investigate the involvement of sRNAs like SrbA in regulating biofilm components such as polysaccharide secretion, regulation of pili and flagella, as well as regulatory effects on metabolic pathways represented in the list of putative mRNA targets. sRNA regulation might have a significant role in these complex responses that are regulated by subtle changes in environmental conditions. Indeed, sRNA regulation has already been shown to be involved in pathways important for regulating biofilms in *P*. *aeruginosa* [55,56,57]. Taken together these putative target searches assist in guiding future work to investigate the specific regulatory involvement this sRNA has and how that contributes to the phenotypes observed in the *srbA* deletion.

## Conclusions

Transcriptomic data and deep-sequencing have provided a vastly greater resolution of expression profiles in pathogens. These technologies have also provided new perspectives on previously underappreciated regulatory mechanisms such as sRNAs. However, determining how sRNAs fit into regulatory networks and what roles they have in the cell is still poorly understood. In this work, we demonstrated that the SrbA is important for biofilm growth in *P*. *aeruginosa*. We also determined that the expression of SrbA has a role in *P*. *aeruginosa* having full pathogenicity when infecting *C*. *elegans*. It is possible that SrbA is interacting with multiple targets that result in the phenotypic effects observed based on the 61 putative mRNA targets found here and that trans-sRNAs are characterized by having a large number of diverse mRNA targets [25,26]. It is important that continued work builds on recent transcriptomic studies to characterize the functional roles of novel, regulatory sRNAs found in *P*. *aeruginosa*. This will help us to not only gain a better understanding of basic biology of bacteria but also infectious states of pathogenic bacteria where nuances of regulation contribute to continued difficulty in treating infections due to biofilm adaptation to the stress of the host environment.

## Supporting information

S1 Fig PCR amplification of the chromosomal locus of *srbA*. A region of 1 kilobase pairs (kbp) in length containing the *srbA* gene was amplified. Wildtype strains (WT and WT2) gave a 1 kbp amplification product while respective *srbA* deletion strains (Δ and Δ2) produced a product 600 bp in length reflecting the 300 bp chromosomal deletion of *srbA*. WT and Δ were used for the entirety of this study. NTC stands for “non-template control”. The values for the 1 kbp ladder are base pair lengths.
(TIF)

S2 Fig Expression of SrbA is restored in a complementation strain. Through use of RT-qPCR, SrbA expression was confirmed to be lost in the deletion strain (Δ) for biofilm cultures. Re-introduction of SrbA expression from a plasmid in a complementation strain (+) restored wildtype levels of expression (WT). Three biological replicates are represented in the graph and error bars are the standard error of the mean.
(TIF)

S3 Fig No polar effects were observed in expression of the gene *aceA* comparing the *srbA* deletion strain to the wild-type strain. RT-qPCR was used to demonstrate there was no change greater than 2-fold in gene expression of *aceA* when comparing the SrbA mutant and wild-type *P*. *aeruginosa*. This indicates that deletion of *srbA* does not have a major effect on the expression of *aceA* downstream. Results presented are from 3 biological replicates and error bars are the standard error of the mean.
(TIF)

S1 Table Primers used in this study. Gene names or PA14 gene designations are provided.
(DOCX)
