# Supplementary figures and images for: A novel small RNA is important for biofilm formation and pathogenicity in Pseudomonas aeruginosa
Source: PLoS One. 2017 Aug 3;12(8):e0182582. doi: 10.1371/journal.pone.0182582 (PMC5542712; doi:10.1371/journal.pone.0182582)

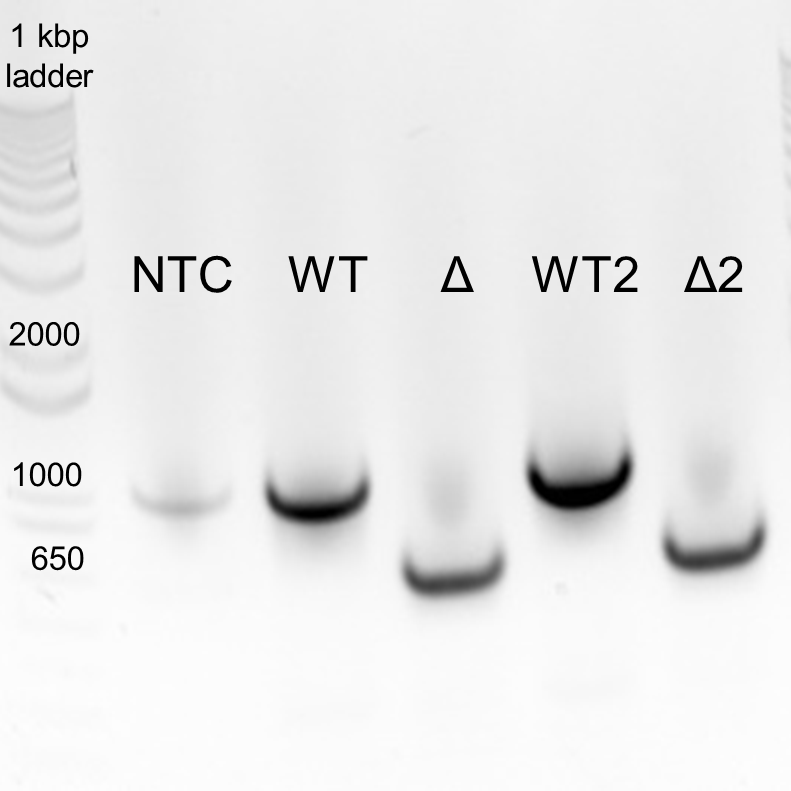

Supplement: S1 Fig — A region of 1 kilobase pairs (kbp) in length containing the srbA gene was amplified. Wildtype strains (WT and WT2) gave a 1 kbp amplification product while respective srbA deletion strains (Δ and Δ2) produced a product 600 bp in length reflecting the 300 bp chromosomal deletion of srbA. WT and Δ were used for the entirety of this study. NTC stands for “non-template control”. The values for the 1 kbp ladder are base pair lengths. (TIF) [file pone.0182582.s001.tif]

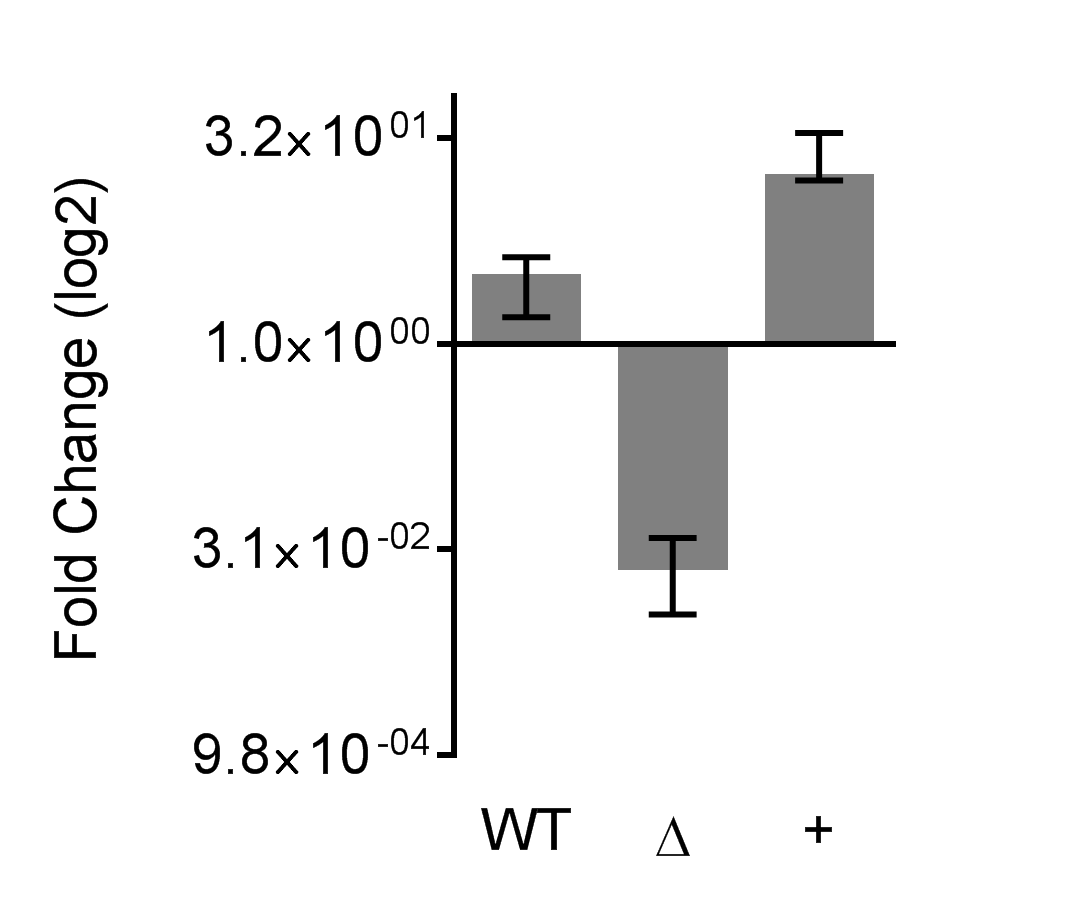

Supplement: S2 Fig — Through use of RT-qPCR, SrbA expression was confirmed to be lost in the deletion strain (Δ) for biofilm cultures. Re-introduction of SrbA expression from a plasmid in a complementation strain (+) restored wildtype levels of expression (WT). Three biological replicates are represented in the graph and error bars are the standard error of the mean. (TIF) [file pone.0182582.s002.tif]

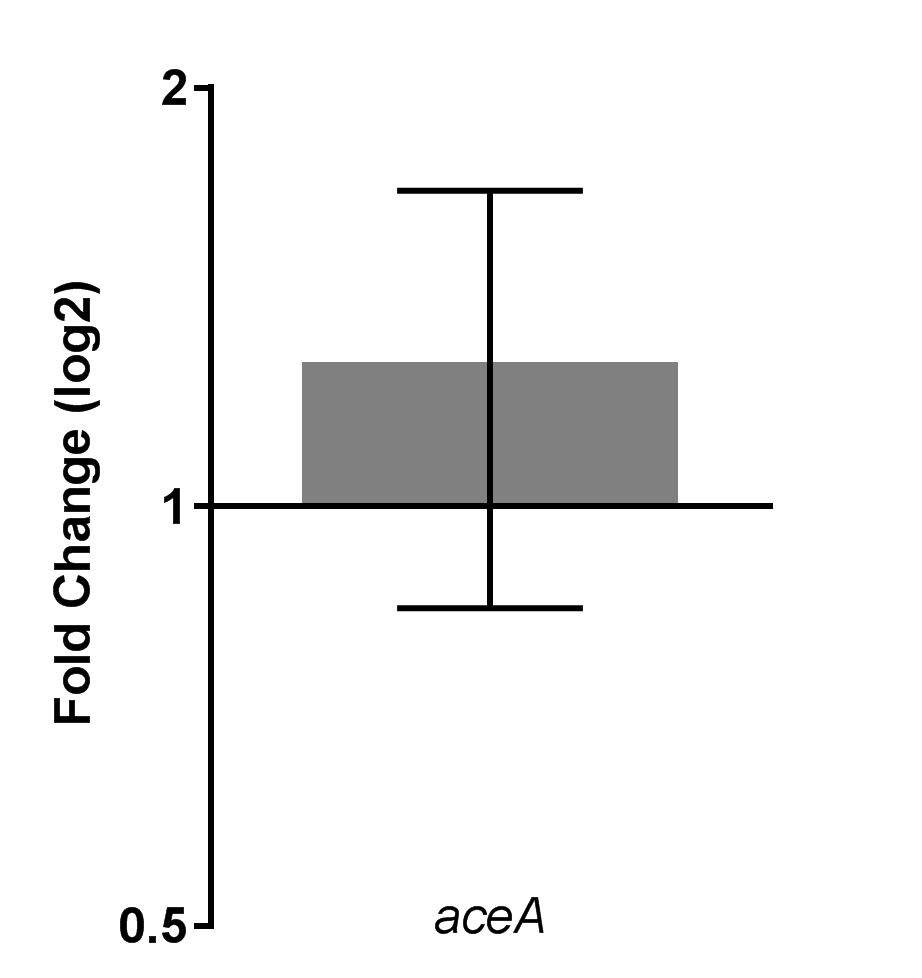

Supplement: S3 Fig — RT-qPCR was used to demonstrate there was no change greater than 2-fold in gene expression of aceA when comparing the SrbA mutant and wild-type P. aeruginosa. This indicates that deletion of srbA does not have a major effect on the expression of aceA downstream. Results presented are from 3 biological replicates and error bars are the standard error of the mean. (TIF) [file pone.0182582.s003.tif]
