# Supplementary material for: A novel small RNA is important for biofilm formation and pathogenicity in Pseudomonas aeruginosa
Source: PLoS One. 2017 Aug 3;12(8):e0182582. doi: 10.1371/journal.pone.0182582 (PMC5542712; doi:10.1371/journal.pone.0182582)
Supplement: S1 Table — Gene names or PA14 gene designations are provided. (DOCX) [file pone.0182582.s004.docx]

**S1 Table. Primers used in this study. Gene names or PA14 gene designations are provided.**

| **Primer** | **Sequence (5’ to 3’)** |
| --- | --- |
| *srbA* (RT-qPCR, forward) | ACTCGCTGTCGAGTCTTTCG |
| *srbA* (RT-qPCR, reverse) | TGCGACTGGAACGAAGTTGT |
| *rpoD* (RT-qPCR, forward) | TCCTGGCCGACTACAATCGC |
| *rpoD* (RT-qPCR, reverse) | TTGACCGGCTCCACCTCTTC |
| *aceA* (RT-qPCR, forward) | TACGGCATTGATCTTGGCGA |
| *aceA* (RT-qPCR, reverse) | GTGTCCGACGAGAAGCAGTG |
| *tag* (RT-qPCR, forward) | GTTCCTCTGGTCCTTCGTCG |
| *tag* (RT-qPCR, reverse) | CTCGGCTTCCGGGGTAATC |
| *pcaK* (RT-qPCR, forward) | CAAGGTGATCGGCCTGTTCTA |
| *pcaK* (RT-qPCR, reverse) | CAGGGTTGCCAGTAGGGTG |
| PA14_03560 (RT-qPCR, forward) | CCTGTCCCTGCAATACCTGG |
| PA14_03560 (RT-qPCR, reverse) | TCCTGGCCGAGATAGTCCC |
| *nirJ* (RT-qPCR, forward) | GACATCTGCAACGGCAACAC |
| *nirJ* (RT-qPCR, reverse) | TCCTGATCGCTGAGGTAGCA |
| PA14_08310 (RT-qPCR, forward) | GTTACCGGTACTGGCACACA |
| PA14_08310 (RT-qPCR, reverse) | TCGTACCAACAACCATCGGG |
| *secE* (RT-qPCR, forward) | TCGCGTTCTCGGTATTCTCG |
| *secE* (RT-qPCR, reverse) | TAAAGAAGGCCTGCCCCTTG |
| *hpaA* (RT-qPCR, forward) | CCGAAGATTTCCGTGCCTCT |
| *hpaA* (RT-qPCR, reverse) | GTAGATCTCACGGTCGTCGC |
| PA14_11790 (RT-qPCR, forward) | CGAGGAAACCAAGAACCCCA |
| PA14_11790 (RT-qPCR, reverse) | ACCGACGACCAGAATCAGTG |
| PA14_12740 (RT-qPCR, forward) | TCAGTTTGCCGATCCCGAG |
| PA14_12740 (RT-qPCR, reverse) | GCTATCGCTGAAATCGCCATC |
| PA14_12910 (RT-qPCR, forward) | ACAGTGGACTGCTGGCG |
| PA14_12910 (RT-qPCR, reverse) | CAGCAGACGGGCTTCCAG |
| PA14_12920 (RT-qPCR, forward) | TCGCTACCCCGTTCGTTTC |
| PA14_12920 (RT-qPCR, reverse) | TGACGATCTTCACCTTGGACG |
| *recJ* (RT-qPCR, forward) | GAGCATCCAGGAGTTCCACC |
| *recJ* (RT-qPCR, reverse) | GAACATCGGCTCGGGAAAGT |
| *lasB* (RT-qPCR, forward) | ACGCTTGACCTGTTGTTCGT |
| *lasB* (RT-qPCR, reverse) | GGGAGTTTGGACACGTCGAT |
| PA14_16870 (RT-qPCR, forward) | TCGATCCGAACCAGAAGCAC |
| PA14_16870 (RT-qPCR, reverse) | ATGGGTGGAGATGATCACGATG |
| PA14_17650 (RT-qPCR, forward) | AGGTGGAAGGCAAGCGAC |
| PA14_17650 (RT-qPCR, reverse) | GTAGGGACTCTTGCGGAACAT |
| *fruI* (RT-qPCR, forward) | GCCGATTTCCCACGAAGAGA |
| *fruI* (RT-qPCR, reverse) | CAACTGGGTCTCGAGGATCTG |
| *mexQ* (RT-qPCR, forward) | ACCGATCATGGCGATTACCTC |
| *mexQ* (RT-qPCR, reverse) | ATAGAACTCGCCCTGCAGAC |
| PA14_20290 (RT-qPCR, forward) | ACTCCTACCTACTCCAGCCG |
| PA14_20290 (RT-qPCR, reverse) | ACTTCTGCGATCTGCTCACG |
| PA14_21750 (RT-qPCR, forward) | GATCGTCCCGGAATTCACCA |
| PA14_21750 (RT-qPCR, reverse) | TCCAGATTCACATGCCGGAC |
| PA14_23090 (RT-qPCR, forward) | GCCGCTTCAAGCTGTTTCC |
| PA14_23090 (RT-qPCR, reverse) | GATATCGGGGAATGGTCCCG |
| *serC* (RT-qPCR, forward) | ACGACTACTTCGCCATTCCG |
| *serC* (RT-qPCR, reverse) | GTCTCGTTGGAGGCGTAGTG |
| PA14_25020 (RT-qPCR, forward) | GAGCAGACGGTGATCGACAA |
| PA14_25020 (RT-qPCR, reverse) | GCCGAGATAGCTGAGCACAT |
| PA14_25400 (RT-qPCR, forward) | CAGGGGCTTCACGTCTCG |
| PA14_25400 (RT-qPCR, reverse) | AAGTCTGTGATCAGGCTGTCC |
| PA14_26810 (RT-qPCR, forward) | GGCCTGTCGATCAGCTACAA |
| PA14_26810 (RT-qPCR, reverse) | GCTGATGAGGAAGCGGGTT |
| PA14_28300 (RT-qPCR, forward) | TCTCGACGATCTACCTGGTCT |
| PA14_28300 (RT-qPCR, reverse) | GAAGTCGATGAACAGCCGGT |
| PA14_29230 (RT-qPCR, forward) | AGATCAAGGTTGGACAGCTCG |
| PA14_29230 (RT-qPCR, reverse) | CGATCCACTTGATGGCCTCT |
| PA14_29260 (RT-qPCR, forward) | AACTGGTTTTCGATGGCGGA |
| PA14_29260 (RT-qPCR, reverse) | TCCGGTGCTGGCACTTTATT |
| PA14_31030 (RT-qPCR, forward) | GCAATACGAATGTGGGCGAC |
| PA14_31030 (RT-qPCR, reverse) | CACGCAGGTCGATCCATACA |
| PA14_32750 (RT-qPCR, forward) | CTGGAGTTCCACCACAACCT |
| PA14_32750 (RT-qPCR, reverse) | AGTCGAGGAACCAGAACATCG |
| PA14_33190 (RT-qPCR, forward) | TGCAACGCTATCGGACGTT |
| PA14_33190 (RT-qPCR, reverse) | ATGTCCGCCCTCCTCTTCT |
| *pslL* (RT-qPCR, forward) | GGGCATCCTGATCATCGTCTT |
| *pslL* (RT-qPCR, reverse) | GAACGGAGAGAGCAACGGAT |
| *cynS* (RT-qPCR, forward) | ACGAGATGCTGCAAGTCTACG |
| *cynS* (RT-qPCR, reverse) | GAAGTTGATCGCGCTGATGATG |
| PA14_38090 (RT-qPCR, forward) | TGCTGTTCTCGACCAATCCC |
| PA14_38090 (RT-qPCR, reverse) | TCGCTTCGTAGTACTTGTCGATG |
| PA14_38290 (RT-qPCR, forward) | GCTGGAGGAACTGGACATCG |
| PA14_38290 (RT-qPCR, reverse) | CCCAGTCGCGGATGACATAG |
| PA14_40260 (RT-qPCR, forward) | ATACCGGTACCACGCAACTG |
| PA14_40260 (RT-qPCR, reverse) | ACGACCTGTACCGTGTTGTC |
| *modA* (RT-qPCR, forward) | GGCCTACCAGTTCGTTTCCA |
| *modA* (RT-qPCR, reverse) | GGTTGCGACTTTACCATCCTTG |
| *pscU* (RT-qPCR, forward) | TGAAGCGCGAGTACAAGGAG |
| *pscU* (RT-qPCR, reverse) | TGCTCGATTGCAGTTCCTGA |
| PA14_43710 (RT-qPCR, forward) | GAGGTCTATCACAGCGCCTT |
| PA14_43710 (RT-qPCR, reverse) | ACAGCCAAGCCAGTACCAC |
| *fliK* (RT-qPCR, forward) | GACGCCTGATGTAAAGCCCA |
| *fliK* (RT-qPCR, reverse) | CGGAGAAGCTGGAAGTCTTGT |
| PA14_46530 (RT-qPCR, forward) | AGCCCAGGATGCACAATCTT |
| PA14_46530 (RT-qPCR, reverse) | CAAGCGCAGGCATGATGTAG |
| *ilvA2* (RT-qPCR, forward) | GGCGATCAAGGACATCTACGA |
| *ilvA2* (RT-qPCR, reverse) | ACTTCTTGATCCCCGCTACG |
| *cobT* (RT-qPCR, forward) | CTGACCAACCTGCTGCTCT |
| *cobT* (RT-qPCR, reverse) | AGCTCGCCGAGACATTCC |
| PA14_48010 (RT-qPCR, forward) | CAACATGGTCTACGGCAACG |
| PA14_48010 (RT-qPCR, reverse) | CCTTGAGCGAACCGGTGAA |
| *flgJ* (RT-qPCR, forward) | CGGTGAGCCTGTCGAAGAAT |
| *flgJ* (RT-qPCR, reverse) | CCTGGAGCCTTGCTTCATCT |
| *phnB* (RT-qPCR, forward) | CGATGCCGATGGTGAGATCAT |
| *phnB* (RT-qPCR, reverse) | GTGAGAATCGACTCGGGATGG |
| *acsA* (RT-qPCR, forward) | GCCCTGACCAACCCTGAAA |
| *acsA* (RT-qPCR, reverse) | TGGTTCCACTTGATCTCCGC |
| PA14_53780 (RT-qPCR, forward) | CTACTTCCCGGTGATGGTCTG |
| PA14_53780 (RT-qPCR, reverse) | CCCATCCCCAACGGTGATT |
| PA14_54040 (RT-qPCR, forward) | TCGCTGCAGATGGTGATGTT |
| PA14_54040 (RT-qPCR, reverse) | GGAATGGTCTTGGTCGGGTT |
| *hxcR* (RT-qPCR, forward) | CAGCGCCTACTCCGATACC |
| *hxcR* (RT-qPCR, reverse) | GGATATCGTCCATCAGGCGG |
| *nemO* (RT-qPCR, forward) | GCTCGAACTCGACGAGAACT |
| *nemO* (RT-qPCR, reverse) | ACGAAGCTCTTCCAGCCTTG |
| *recC* (RT-qPCR, forward) | GTGAATGCGTTCTTCCAGCAG |
| *recC* (RT-qPCR, reverse) | TTCGAAAGGCTCTTCGTCGT |
| *ampG* (RT-qPCR, forward) | CCATCACCCTCGACAACTTCA |
| *ampG* (RT-qPCR, reverse) | CGGAGAACTTCAGGTTGGTCA |
| PA14_59010 (RT-qPCR, forward) | CCTTGCCGAACGACTGTTTG |
| PA14_59010 (RT-qPCR, reverse) | CTCTCGATGCGCTTTTCGTG |
| PA14_59030 (RT-qPCR, forward) | TTCGCTCAAGCAGAAGGCT |
| PA14_59030 (RT-qPCR, reverse) | AGAAATGAAGATCGAGGCGCA |
| *dtd* (RT-qPCR, forward) | ATAGAAATACCGGCCGTGAGC |
| *dtd* (RT-qPCR, reverse) | ACCGTGGTATGGCTCAGATG |
| PA14_61990 (RT-qPCR, forward) | TTCGGCAAGTACAGCATGGA |
| PA14_61990 (RT-qPCR, reverse) | GCAGCATCAGTTCGAGGAGAA |
| PA14_64280 (RT-qPCR, forward) | GTGATCCGCCATCTCTACGG |
| PA14_64280 (RT-qPCR, reverse) | ACCAGTTGGATCAGCACCAG |
| PA14_64530 (RT-qPCR, forward) | CCACCCTGACCGCCAAC |
| PA14_64530 (RT-qPCR, reverse) | GCAGTTCGACGCTGGACA |
| PA14_66380 (RT-qPCR, forward) | GCAAAGGTCTCAACGAGCG |
| PA14_66380 (RT-qPCR, reverse) | GGTCACGGTGAGGAATACCG |
| PA14_66510 (RT-qPCR, forward) | CTGGCATTACTACGGCTGGA |
| PA14_66510 (RT-qPCR, reverse) | CAGGTGCAGCGAGACCAATA |
| PA14_68670 (RT-qPCR, forward) | AACCCTACTACCGCCTGGA |
| PA14_68670 (RT-qPCR, reverse) | CAATCGGTGAACGAGCCCA |
| *adhA* (RT-qPCR, forward) | AATTCTGCCGAAAAACGTCGAA |
| *adhA* (RT-qPCR, reverse) | GTCTGCTTGAGCCCCTTGT |
| *srbA* deletion 1 | GGTGGTGAATTCAAGGAGTTGTACAGGTCGCC |
| *srbA* deletion 2 | GGTGGTGGATCCTTTGAGTGGGTCAGTCGGTG |
| *srbA* deletion 3 | GGTGGTGGATCCCCGTCGTCAAACGTTTTGTAGT |
| *srbA* deletion 4 | GGTGGTGTCGACGTGATCCCGCTCAACTACCG |
| *srbA* complementation (forward) | GGTGGTGTCGACATCAGGGGCTCTGAAACGAC |
| *srbA* complementation (reverse) | GGTGGTGAATTCCACCGACTGACCCACTCAAA |
| *srbA* chromosomal flank (forward) | ACCATCCAACCGGACAGGTA |
| *srbA* chromosomal flank (reverse) | GGTGTCAGCGAACAGAACCA |
